# Cerebral aneurysms at major arterial bifurcations are associated with the arterial branch forming a smaller angle with the parent artery

**DOI:** 10.1038/s41598-022-09000-7

**Published:** 2022-03-24

**Authors:** Bu-Lang Gao, Hong Hao, Weili Hao, Chun-Feng Ren, Lei Yang, Yongfeng Han

**Affiliations:** 1 Department of Medical Research and Neurosurgery, Shijiazhuang People’s Hospital, 9 Fangbei Road, Shijiazhuang, 050011 Hebei China; 2 Department of Laboratory Analysis, Zhengzhou University First Affiliated Hospital, Zhengzhou, China

**Keywords:** Computational biology and bioinformatics, Neuroscience, Diseases, Medical research, Neurology

## Abstract

Currently, the relationship of bifurcation morphology and aneurysm presence at the major cerebral bifurcations is not clear. This study was to investigate cerebral arterial bifurcation morphology and accompanied hemodynamic stresses associated with cerebral aneurysm presence at major cerebral arterial bifurcations. Cerebral angiographic data of major cerebral artery bifurcations of 554 anterior cerebral arteries, 582 internal carotid arteries, 793 middle cerebral arteries and 195 basilar arteries were used for measurement of arterial diameter, lateral and bifurcation angles and aneurysm deviation. Hemodynamic stresses were analyzed using computational fluid dynamic simulation. Significantly (*P* < 0.001) more aneurysms deviated toward the smaller branch and the smaller lateral angle than towards the larger branch and larger lateral angle at all four major bifurcations. At the flow direct impinging center, the total pressure was the greatest while the dynamic pressure, wall shear stress (WSS), vorticity and strain rate were the least. Peak 1 and Peak 2 were located on the branch forming a smaller and larger angle with the parent artery, respectively. The dynamic pressure (175.4 ± 18.6 vs. 89.9 ± 7.6 Pa), WSS (28.9 ± 7.4 vs. 15.7 ± 5.3 Pa), vorticity (9874.6 ± 973.4 vs. 7237.8 ± 372.7 1/S), strain rate (9873.1 ± 625.6 vs. 7648.3 ± 472.5 1/S) and distance (1.9 ± 0.8 vs. 1.3 ± 0.3 mm) between the peak site and direct flow impinging center were significantly greater at Peak 1 than at Peak 2 (*P* < 0.05 or P < 0.01). Moreover, aneurysms deviation and Peak 1 were always on the same side. In conclusion, the branch forming a smaller angle with the parent artery is associated with abnormally enhanced hemodynamic stresses to initiate an aneurysm at the bifurcation apex.

## Introduction

Cerebral aneurysms occur in 2–4% of the general population, and the most dreaded complication of aneurysms is rupture^1^. Despite advances in microinvasive and invasive surgical techniques and post-operative management, the mortality and morbidity of aneurysm rupture remain high. Increasing evidence shows that enhanced hemodynamic insult is the initiating factor for aneurysm formation, and hemodynamic stress-induced inflammatory progress is the leading factor in the pathogenesis of aneurysm formation^2–5^. However, aneurysm initiation site which experiences abnormal hemodynamic stresses and inflammatory progress remain unclear even though some destructive remodeling of arterial wall has been observed to associate with aneurysm initiation^4^.

Cerebral arterial bifurcation apexes are the most frequent location of aneurysm formation, and the bifurcation wall is exposed to the maximal hemodynamic stresses, which are not only affected by arterial diameter, but also by the bifurcation angle formed between two branches and the lateral angles formed between the parent artery and one branch artery. Our previous studies have shown that both anterior communicating artery and basilar apex (BA) aneurysms are not only deviated to the smaller branch forming a smaller lateral angle with the parent artery but also associated with wider bifurcation angles and narrower lateral angles^6,7^. Enlarged anterior cerebral artery (ACA) bifurcation angles possibly induce abnormally enhanced hemodynamic stresses to initiate aneurysms^8^. In clinical setting, stent-mediated coiling of wide-necked aneurysms significantly increases the lateral angle formed between the parent artery and stented daughter branch but decreases the bifurcation angle and the pressure at the bifurcation apex. These changes in the arterial angles subsequently displace and attenuate the flow impingement zone at the neck of bifurcation aneurysms, leading to decreased wall shear stress (WSS) and total pressure at the bifurcation as well as reduced damage to the arterial wall caused by the WSS and total pressure^9–11^.

Better understanding of the relationship between hemodynamic stresses and arterial bifurcation morphology at the aneurysm initiation site is crucial for development of new preventive and therapeutic strategies. It has been reported that the region enclosed by the aneurysm neck is the aneurysm initiation site^12^, however, the hemodynamic stresses within this area have not been specified to induce an aneurysm. We hypothesized that the specific hemodynamic stresses at the location of aneurysm neck were associated with presence of saccular aneurysms at four major cerebral arterial bifurcations, including the internal carotid artery (ICA), ACA, middle cerebral artery (MCA) and BA. This study was consequently performed to analyze the hemodynamic stresses before and after aneurysm formation at the bifurcation apex and possible factors affecting arterial diameter, lateral and bifurcation angles by using patients’ specific cerebral angiographic data coupled with computational fluid dynamic (CFD) analysis so as to elucidate the possible arterial morphology and hemodynamic stresses favoring aneurysm formation.

## Materials and methods

### Subjects

This retrospective case–control one-center study was approved by the ethics committee of Shijiazhuang People’s Hospital, and all patients had given their signed informed consent to participate. All methods were performed in accordance with the relevant guidelines and regulations. Between March 2004 and February 2015, consecutive patients who had three-dimensional digital subtraction angiography in our hospital were reviewed in this study. All volumes showing a clear view of major arterial bifurcations were included, and those with unclear imaging data were excluded. CFD analysis was performed in 318 patients with saccular cerebral aneurysms at the major cerebral arterial bifurcations, including the ICA (n = 35), ACA (n = 115), MCA (n = 109) and BA (n = 59) bifurcations. The symptoms of these patients included subarachnoid hemorrhage, headache, confusion, face numbness, double vision and nonspecific neurological symptoms. Control none-aneurysmal subjects with no cerebral aneurysms were also evaluated in 1806 patients (439 ACA, 547 ICA, 136 BA and 684 MCA bifurcations) who had digital subtraction angiography for suspected cerebrovascular diseases for comparison with the aneurysmal group (Table 1). Data on patients’ age, gender, symptoms and aneurysm status were collected from a prospectively maintained database. There was no statistically (*P* > 0.05) significant difference in the mean age or gender percentage between the aneurysmal and control groups.

**Table 1:** Baseline characteristics of patients.

	ACA bifurcations (n = 554)	ICA bifurcations (n = 582)	BA bifurcations (n = 195)	MCA bifurcations (n = 793)
**Gender**				
Female	335	403	110	554
Male	219	179	85	239
Mean age in years	52.6 ± 14.5 (11–92)	55.4 ± 13.9 (11–92)	53.0 ± 14.5 (18–82)	55.3 ± 13.9 (11–92)
**Bifurcations character**				
Normal	439	547	136	684
With aneurysms	115	35	59	109
**Aneurysm type**				
Type C	85	17	52	68
Type D	30	16	7	41
**φ2 formation**				
Between D1 and D2	75 (65.2%)***	28 (80%)***	38(64.4%)*	99(90.8%)***
Between D1 and D3	40 (34.8%)	7(20%)	21(35.6%)	10(9.2%)

Data are shown as mean ± standard deviation.

*ACA* anterior cerebral artery, *ICA* internal carotid artery, *BA* basilar artery, *MCA* middle cerebral artery.

Type C, the parent vessel centerline crosses the aneurysm neck, and type D, the parent vessel centerline does not cross the aneurysm neck. D1, D2 and D3 indicate the diameter of the parent vessel, smaller and larger daughter branch, respectively. *P < 0.05 and ***P < 0.001 indicate significant differences of angle φ2 formed between D1 and D2, D1 and D3, respectively.

### Measruement of vascular diameter, angle and stresses

**Figure 1:**
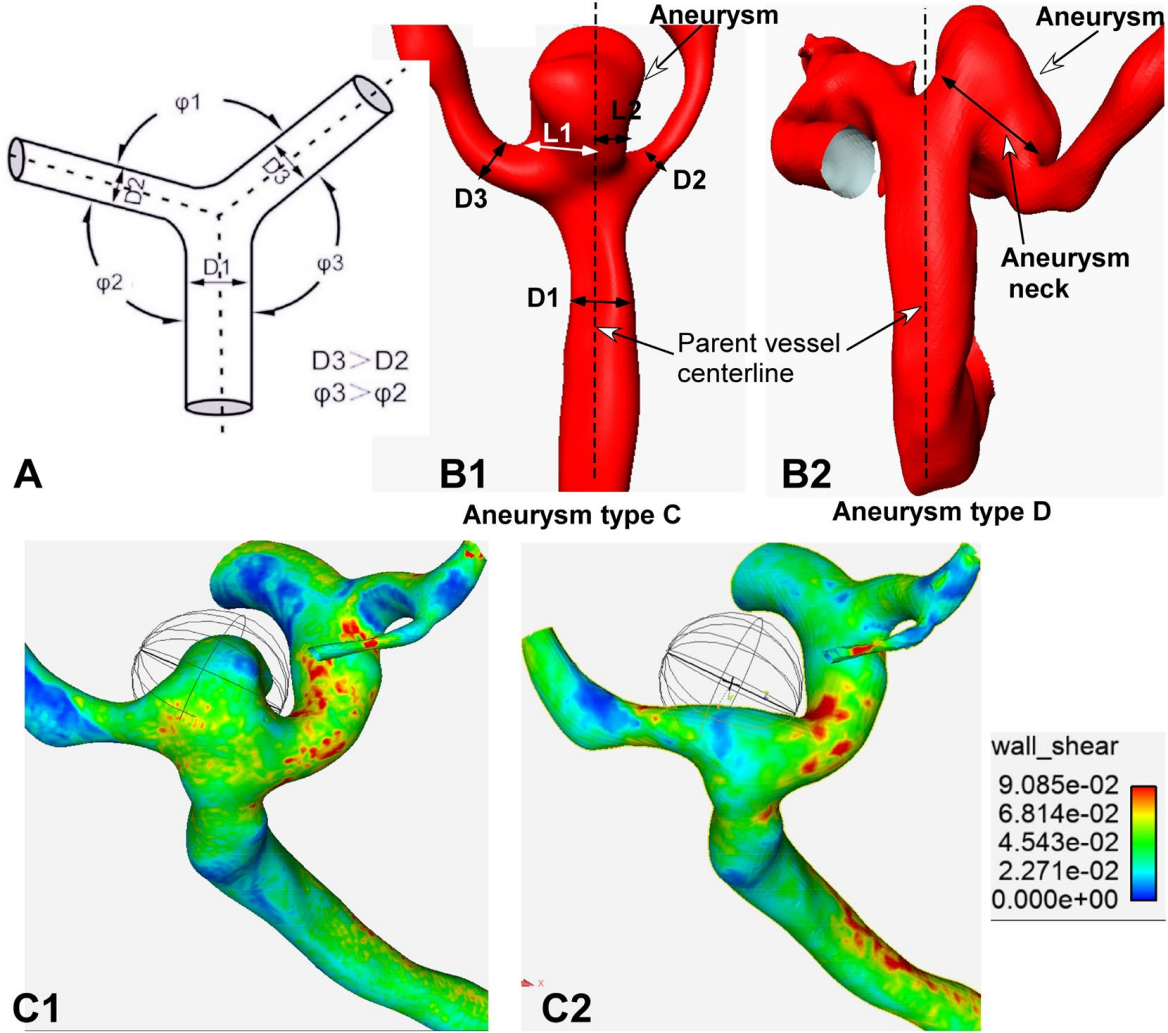
Measurement of arterial morphology. (**A**) Schematic drawing shows the definition of D1–D3, and φ1–φ3. The diameter of the parent artery is D1, and the bifurcation angle between branching arteries is φ1. The larger lateral angle and larger daughter branch are defined as angle φ3 and D3, respectively, whereas the samller contralateral lateral angle and branch artery are termed as φ2 and D2, respectively, with D3 > D2 and φ3 > φ2. (**B1**–**B2**) Types of aneurysms based on the aneurysm neck. (**B1**) Classical aneurysms (type C aneurysms) are those in which the parent vessel centerline passes through the aneurysm neck. The aneurysm neck is divided into 2 sections: L1 and L2. If L1 is greater than L2, the aneurysm deviates towards the L1 side. Otherwise, it dviates towards the other side. (**B2**) Type D aneurysms are those with a deviating neck in which the parent vessel centerline does not pass through the aneurysm neck. (**C1**, **C2**) Sphere sampling is shown. (**C1**) A sphere is used to sample the hemodynamic stresses on the aneurysm dome. (**C2**) A sphere is used to sample the hemodynamic stresses on the aneurysm initiation site after virtual aneurysm removal.

Three-dimensional rotational angiography data were reconstructed for surface rendering by using Amira software (version 5.2.2, Visage Imaging, San Diego, CA, USA). The vascular diameter at the bifurcations was measured in the way similar to the approach used by Ingebrigtsen et al.^13^ and our previous studies^6,7^. The parent vessel diameter was defined as D1, and those for the smaller and larger vessels as D2 and D3, respectively (Fig. 1A). The lateral angles (smaller one was defined as φ2, and the contralateral larger one as φ3) at the four major cerebral arterial bifurcations were measured. As reported in our previous reports^6,7^, the angle was measured by use of 3 dots after the central point was placed at the tip of the bifurcation in line with the central axis of one daughter artery, and the other 2 dots marked the central axis of the parent artery and the other daughter branch (Fig. 1A). A sphere was used to sample the hemodynamic stresses on the aneurysm dome and at the aneurysm initiation site after virtual aneurysm removal (Fig. 1C1, C2).

### Aneurysm neck and deviation

According to the neck location of aneurysms, aneurysm necks were classified into two types as previously reported^14^. The neck that was located on the extension of the parent artery centerline was defined as the classic neck (type C); when it was not, it was termed as the deviating neck (type D). For neck type C, the measurement method of aneurysm deviation was in line with our previous study^6^ as shown in Fig. 1B1. Briefly, the length of neck was divided into two sections by the parent artery centerline, defined as L1 and L2. If L1 was greater than L2, aneurysm deviation was towards the L1 side. If L2 was greater than L1, the aneurysm was deviated towards L2. For neck type D, aneurysm deviation was evaluated based on the location of the aneurysm (Fig. 1B2).

### CFD analysis

CFD analysis was performed using three-dimensional cerebral angiographic datasets which were reconstructed for surface rendering with the Amira software. Surface smoothing, virtual removal and surface reconstruction of the aneurysm were all performed with the Meshlab software (Meshlab v1.3.3, Visual computing Lab, ISTI, CNR) similar to a previous report^11^. Finite-volume solution was performed with the Fluent software (version 12.0.16, Ansys, Lebanon, NH, USA) in the laminar double-precision unsteady manner with assumption of constant flow input, rigid nonslip wall conditions, blood density of 1070 kg/m^3^ and blood viscosity of 3.5 cP as previously described^15^. The inflow rate was set at 0.1 m/s, and the outlet pressure was set at zero. Preliminary CFD analysis which was performed at the peak systole using time-dependent runs for five patients demonstrated similar outcomes to the steady-state analysis, and the CFD analysis was consequently conducted using the steady-state mode for minimizing variability.

### Line sampling of vascular hemodynamic stresses

**Figure 2:**
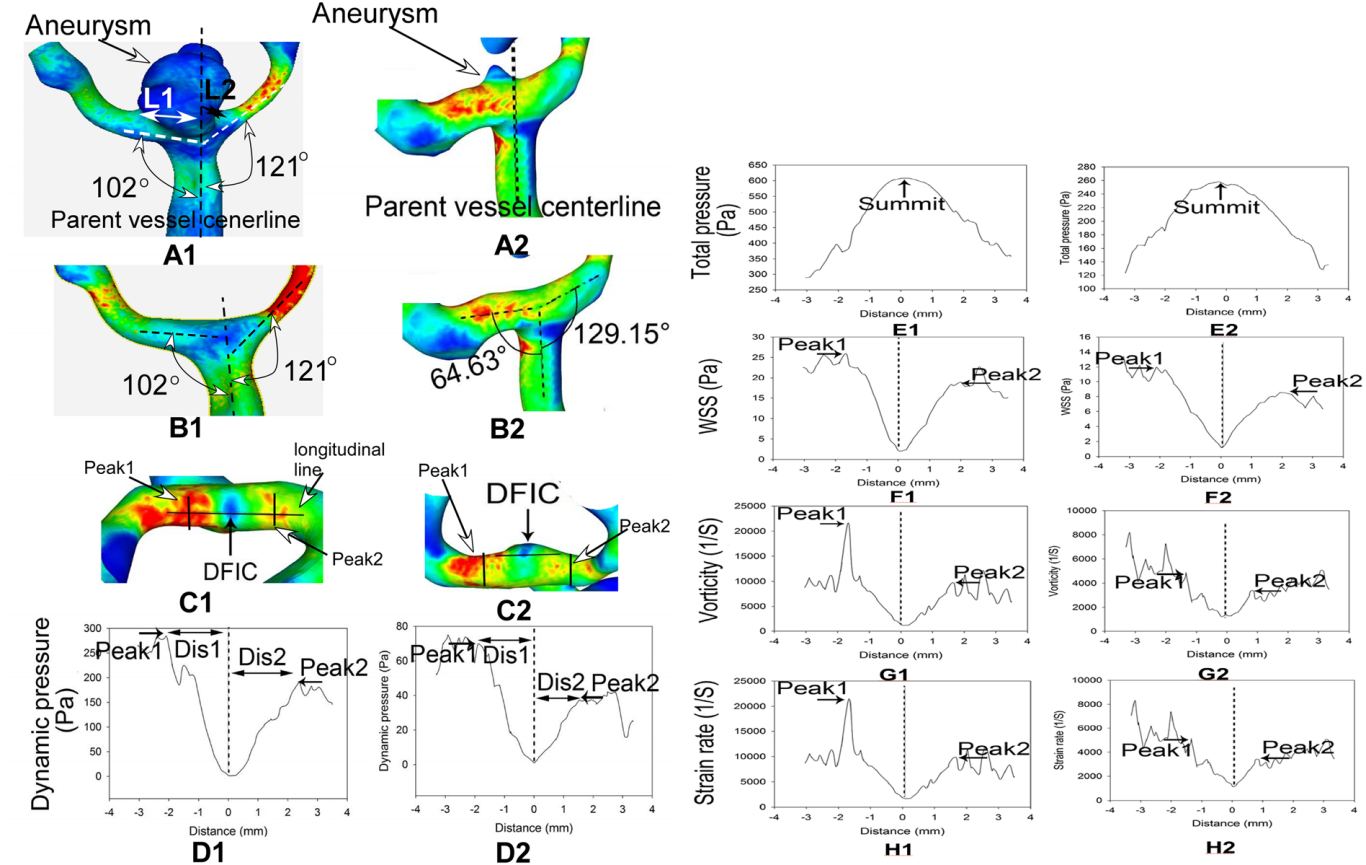
Line sampling of hemodynamic stresses on the bifurcation apex wall. (**A1**–**H1**) An unruputred anterior communicating artery (Acom) aneurysm and sampling on the bifurcation apex after virtual aneurysm removal. (**A1**) The Acom aneurysm was shown. L1 was larger than L2, and the aneurysm deviated towards the L2 side. B1-C1. After the aneurysm was virtually removed (**B1**), a longitudinal line was drawn on the top of the bifurcation apex across the flow direct impinging center (DFIC), Peaks 1 and 2 (**C1**) to sample the hemodynamic stresses including the dynamic pressure, total pressure, wall shear stress (WSS), vorticity, and strain rate. (**D1**–**H1**). The profiles of hemodynamic stresses on the longitudinal line were shown. There were two peaks of stresses on the line: Peaks 1 and 2. Dis represented the distance from the DFIC to Peak 1 or 2. Distance 0 indicated the DFIC where the flow direction was perpendicular to the wall. Beyond Peak 1 or 2, the flow direction was parallel to the arterial wall. (**A2**–**H2**). An unruputred Acom aneurysm in a 580 year-old man. (**A2**) The Acom aneurysm was shown. (**B2**–**C2**) After virtual aneurysm removal (**B2**), a longitudinal line was drawn on the bifurcation apex to smaple the hemodynamic stresses (**C2**). **D2**–**H2**. The profiles of hemodynamic stresses on the longitudinal line were shown, similar to those in (**D1**–**H1**).

**Figure 3:**
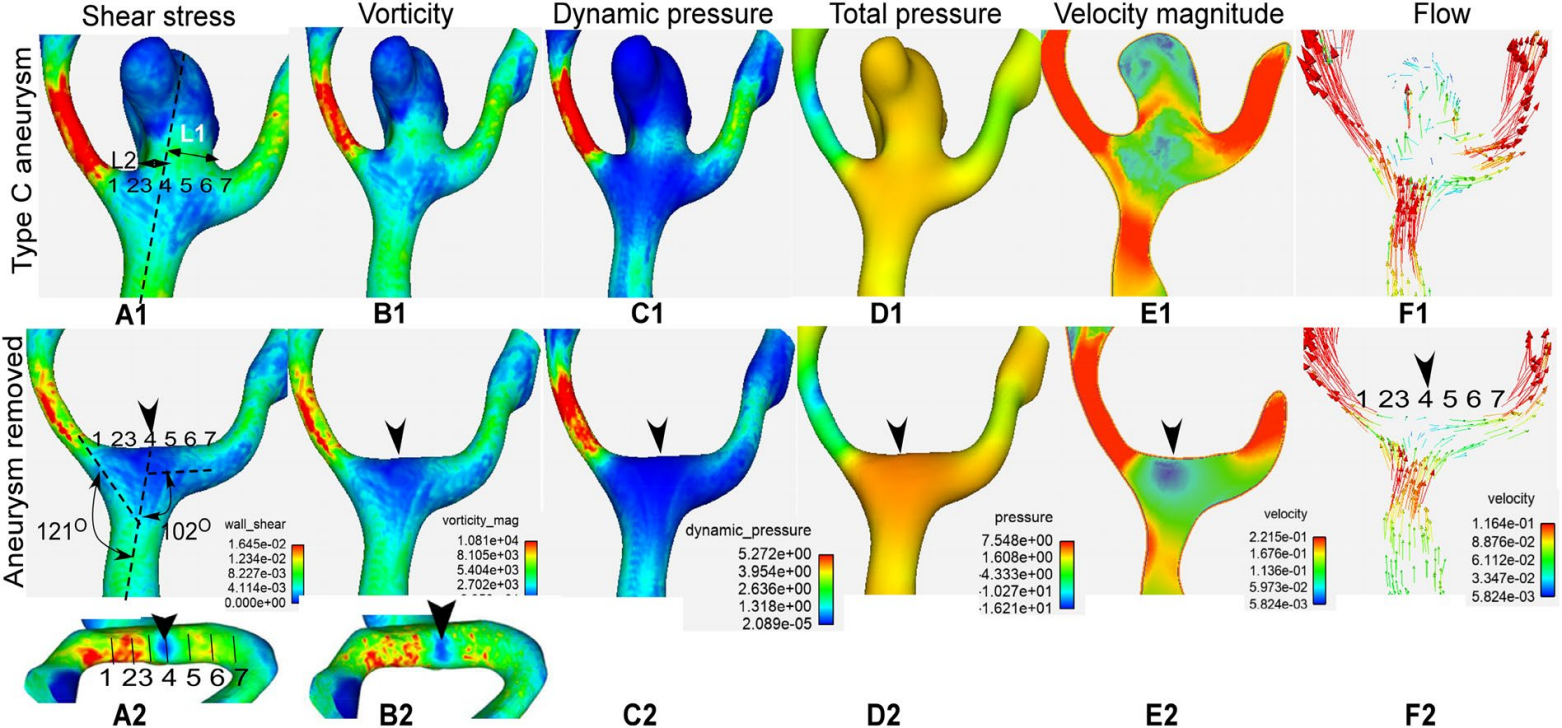
Demonstration of hemodynamic stresses on a type C aneurysm (anterior communicating artery aneurysm) before (**A1**–**F1**) and after (**A2**–**F2**) virtual aneurysm removal. L1 and L2 in A1 represent the length of aneurysm neck divided by the parent vessel axial line, and L1 was greater than L2, indicating deviation of the aneurysm toward the L1 side (**A1**). Seven transverse lines were drawn on the bifurcation apex for sampling hemodynamic stresses after aneurysm removal (**A2**). Transverse line 4 was located at the direct flow impinging center (DFIC, arrowhead), line 1 at the daughter branch forming a smaller lateral angle with the parent vessel, and line 7 at the contralateral branch artery. Lines 1 and 7 were located outside of the aneurysm neck. The shear stress (**A1**, **A2**), vorticity (**B1**, **B2**), dynamic pressure (**C1**, **C2**) and total pressure (**D1**, **D2**) were significantly smaller on the aneurysm dome (**A1**–**D1**) than at the aneurysm initiation site after aneurysm removal (**A2**–**D2**). The dynamic pressure, total pressure and velocity magnitude were significantly smaller inside the aneurysm than in the arterial lumen near the bifurcation apex. Blood flow gets in the aneurysm and two branching arteries before aneurysm removal and hits directly at the direct flow impinging center (DFIC) on the bifurcation apex after aneurysm removal (**E**, **F**). Arrowheads indicate the DFIC.

Post-processing was performed with the Ensight software (Version 10.1; CEI, Apex, NC, USA). One longitudinal and seven transverse lines were drawn for sampling of hemodynamic stress parameters on the bifurcation apex after aneurysm virtual removal (Figs. 2 and 3). The longitudinal line was drawn in the middle of the distal bifurcation apex wall, and the seven transverse lines were drawn according to the WSS profile on the bifurcation apex. The WSS profile in the longitudinal line on the distal bifurcation apex wall had one direct flow impinging center (location of transverse line 4) and two peak values of hemodynamic stress parameters (WSS, dynamic pressure, vorticity and strain rate), with Peak 1 on the smaller branch and Peak 2 on the larger branch. The longitudinal line was through the direct impinging center and two peaks of stresses. Peak 1 was at the location of transverse line 3 while Peak 2 at the transverse line 5. Lines 1 and 2 were located on the distal wall of the smaller branch while lines 6 and 7 on the larger branch, with lines 1 and 7 locating outside the aneurysm profile. Line 2 was located in the middle between lines 1 and 3, whereas line 6 between lines 7 and 5, with a 2 mm distance between these lines. Both lines 2 and 6 were to sample hemodynamic stresses at locations other than the direct flow impinging center and two peaks.

## Statistical analysis

All data were presented as mean ± standard deviation. The JMP statistical software (version 10.01.2, SAS Institute, Cary, NC, USA) was used for statistical analysis. The angle and arterial diameter between two groups were compared using the *t* test. Hemodynamic stress differences at bilateral arterial branches were determined using the *t* test. Aneurysm deviation and smaller lateral angle morphology were analyzed using the chi-square test. *P* value < 0.05 was considered to be statistically significant.

## Results

In ACA bifurcations, D3 was significantly larger than D2 (*P* < 0.001) in both aneurysmal and control groups. Similar differences were also observed in ICA, BA and MCA bifurcations (*P* < 0.001). Moreover, angle φ3 was significantly greater than φ2 in both aneurysmal and control groups in ACA, ICA, BA and MCA bifurcations (*P* < 0.001) (Table 2). The smaller lateral angle φ2 was formed significantly (*P* < 0.001) mostly between the smaller branch (D2) and the parent artery, including 65.2% (n = 75) at the ACA, 80% (n = 28) ICA, 64.4% (n = 38) BA and 90.8% (n = 99) MCA bifurcations. Significantly (*P* < 0.001) few numbers of the smaller lateral angle φ 2 were formed between the larger branch D3 and the parent artery, including 34.8% (n = 40) at the ACA, 20% (n = 7) ICA, 35.6% (n = 21) BA and 9.2% (n = 10) MCA bifurcations.

**Table 2:** Analysis of arterial diameter and lateral angles at major arterial bifurcations.

	ACA bifurcations	ICA bifurcations	BA bifurcations	MCA bifurcations
**All An**				
D2 (mm)	2.1 ± 0.9 (0.8–5.6)	2.7 ± 1.3 (1.0–5.5)	2.8 ± 1.2 (0.9–6.6)	1.6 ± 0.6 (0.9–4.6)
D3 (mm)	2.7 ± 0.9*** (1.4–5.7)	3.4 ± 1.5*** (1.3–7.0)	3.5 ± 1.3*** (1.3–8.0)	2.4 ± 0.9*** (1.3–7.1)
φ2 (°)	94.0 ± 13.2 (60.9–125.1)	65.5 ± 17.9 (36.2–107.2)	91.5 ± 15.6 (49.6–117.7)	76.6 ± 22.9 (38.7–163.3)
φ3 (°)	114.0 ± 13.3*** (79.5–154.4)	134.5 ± 15.0*** (87.6–161.4)	116.6 ± 15.8*** (76.3–147.1)	122.2 ± 24.1*** (58.8–169.5)
**Control subjects**				
D2 (mm)	2.5 ± 1.0 (0.6–5.9)	2.5 ± 1.1 (0.7–7.5)	3.0 ± 1.3 (0.6–6.5)	1.9 ± 0.7 (0.6–5.2)
D3 (mm)	4.1 ± 1.3*** (1.4–10.6)	3.2 ± 1.4*** (1.6–9.4)	3.7 ± 1.5*** (1.2–7.5)	2.5 ± 0.9*** (1.0–8.2)
φ2 (°)	103.4 ± 5.6 (46.1–148.3)	76.4 ± 17.3 (22.2–154.2)	115.5 ± 15.2 (62.4–165.5)	111.3 ± 19.3 (43.7–173.8)
φ3 (°)	126.8 ± 13.6*** (75.2–164.9)	138.0 ± 12.2*** (75.2–172.7)	133.3 ± 11.6*** (103.1–171)	138.2 ± 14.8*** (58.4–212.7)

Data are shown as mean ± standard deviation.

*An* aneurysms, *ACA* anterior cerebral artery, *ICA* internal carotid artery, *BA* basilar artery, *MCA* middle cerebral artery.

***P < 0.001 between D2 and D3 and between φ2 and φ3. AN represents aneurysm. D2 and D3 indicate smaller and larger daughter branch, respectively. φ2 and φ3 represent smaller and larger lateral angle, respectively.

In 115 patients with ACA bifurcation aneurysms, 84.3% (97 cases) and 75.7% (87 cases) aneurysms deviated towards the smaller angle φ2 and the smaller daughter branch D2, respectively, which were significantly (P < 0.001) more than those deviating towards the larger angle φ3 or the larger branch D3 (*P* < 0.001). Furthermore, 67.0% (77 cases) aneurysms deviated towards both the smaller daughter vessel and smaller lateral angle (Table 3).

**Table 3:** Aneurysms deviation.

Bifurcation	Towards D2 (Cases)	Towards D3 (Cases)	Towards φ2 (Cases)	Towards φ3 (Cases)	Towards φ2 and D2 (Cases)
ACA	75.7% (87)***	24.3% (28)	84.3% (97)***	15.7% (18)	67.0% (77)
ICA	77.1% (27)***	22.9% (8)	91.4% (32)***	8.6% (3)	74.3% (26)
BA	62.7% (37)	37.3% (22)	88.1% (52)***	28.8% (17)	57.6% (34)
MCA	81.7% (89)***	18.3% (20)	94.5% (103)***	5.5% (6)	78.0% (91)

*ACA* anterior cerebral artery, *ICA* internal carotid artery, *BA* basilar artery, *MCA* middle cerebral artery.

***P < 0.001 between D2 and D3 and between φ2 and φ3. D2 and D3 indicate smaller and larger daughter branch, respectively. φ2 and φ3 represent smaller and larger lateral angle, respectively.

Similarly, 91.4% (32 cases) and 77.1% (27 cases) ICA bifurcation aneurysms deviated towards the smaller angle φ2 and the smaller branch D2, respectively, significantly (*P* < 0.001) more than thoses deviating towards the larger angle φ3 or larger D3. 74.3% (26 cases) aneurysms deviated towards both the smaller angle φ2 and the smaller branch D2 (Table 2). In MCA bifurcations, significantly more aneurysms deviated towards φ2 (94.5%, 103 cases) and D2 (81.7%, 89 cases) (*P* < 0.001) than aneurysms deviating towards φ3 (5.5%, 6 cases) and D3 (18.3%, 20 cases), respectively, whereas 78.6% (85 cases) aneurysms deviated towards both angle φ2 and D2. In BA bifurcations, 88.1% (52 cases) aneurysms deviated towards the smaller angle φ2, significantly more than aneurysms deviating towards the greater angle φ3 (*P* < 0.001), however, no significant difference (*P* > 0.05) existed in aneurysm deviation towards D3 or D2 (Table 3). 57.6% (34 cases) aneurysms deviated towards both angle φ2 and D2 at BA bifurcations.

On the bifurcation apex wall, there were one direct flow impinging center and two peaks of hemodynamic stresses on the longitudinal line at the bifurcation apex after virtual aneurysmal removal (Fig. 2). At the direct flow impinging center, the total pressure was the greatest while the dynamic pressure, WSS, vorticity and strain rate were the minimal. As blood flowed from the direct flow impinging center towards both branches, the total pressure decreased rapidly but the other parameters increased quickly and reached the peak value (Peak 1 and Peak 2) in the region immediately adjacent to the direct impinging center. Peak 1 and Peak 2 were located on the daughter branch forming a smaller and larger angle with the parent artery, respectively. The dynamic pressure (152.4 ± 16.3 vs. 91.7 ± 9.2 Pa), WSS (19.9 ± 8.5 vs. 13.8 ± 4.8 Pa), vorticity (9869.6 ± 982.2 vs. 7000.1 ± 469.4 1/S), strain rate (9860.1 ± 618.4 vs. 7122.8 ± 416.7 1/S) and distance (1.8 ± 0.9 vs. 1.2 ± 0.5 mm) were significantly greater at Peak 1 than at Peak 2 (*P* < 0.05 or *P* < 0.01, Table 4). Moreover, aneurysms deviation and Peak 1 were always on the same side when using the parent vessel centerline as the reference, regardless of aneurysm type (Fig. 2). The distance from the flow direct impinging center (where the flow direction was perpendicular to the bifurcation apex) to the peak site implied the accelerating area. Dis 1 (the distance between the direct impinging center and Peak 1) was significantly longer than Dis 2 (the distance between the direct impinging center and Peak 2) at the bifurcations for both types C and D aneurysms (*P* < 0.01, Table 4).

**Table 4:** Hemodynamic stresses at Peak 1 and Peak 2 of artery bifurcations (mean ± SD).

	Dynamic pressure (Pa)	Total pressure (Pa)	Wall shear stress (Pa)	Vorticity (1/S)	Strain rate (1/S)	Dis(mm)
Peak1	175.4 ± 18.6** (32.7–552.3)	435.4 ± 26.3 (75.2–1567.3)	28.9 ± 7.4** (4.2–51.4)	9874.6 ± 973.4* (1896.8–24,532.5)	9873.1 ± 625.6* (1934.7–25,686.3)	1.9 ± 0.8** (0.4–5.1)
Peak2	89.9 ± 7.6 (13.7–425.7)	493.5 ± 61.4 (98.3–1748.6)	15.7 ± 5.3 (3.4–42.3)	7237.8 ± 372.7 (1238.3–18,973.8)	7648.3 ± 472.5 (1378.3–21,468.8)	1.3 ± 0.3 (0.3–2.6)

*SD* standard deviation.

Dis represents the distance from the peak site to the flow direct impinging center. Peak1 is on the daughter vessel forming a smaller angle with the parent artery, whereas Peak 2 is on the contralateral daughter artery. *P < 0.05 and **P < 0.01 indicate significant differences in hemodynamic stresses between peak 1 and peak 2.

Among the seven transverse lines on the bifurcation apex wall, transverse line 4 at the direct flow impinging center had the highest total pressure but lowest dynamic pressure, WSS, vorticity and strain rate (Fig. 3 and Table 5). From transverse line 4 to line 1 or 7, the total pressure quickly decreased but the dynamic pressure, WSS, vorticity and strain rate rapidly increased. The dynamic pressure, total pressure, WSS, vorticity and strain rate were significantly (*P *˂ 0.01) greater on line 3 (at Peak 1) than on line 5 (at Peak 2). Significantly (*P* < 0.001) greater hemodynamic stress parameters (dynamic pressure, WSS, vorticity and strain rate) also existed on lines 2 and 1 than on lines 6 and 7, respectively (Table 5).

**Table 5:** Hemodynamic stresses on 7 transverse lines at the ACA bifurcation after virtual aneurysm removal (mean ± SD).

Transverse lines	Dynamic pressure (Pa)	Total pressure (Pa)	WSS (Pa)	Vorticity (1/S)	Strain rate (1/S)
1	229.5 ± 107.1 (78–396)	196.7 ± 50.9 (113–273)	21.1 ± 7.2 (9.7–31.9)	9272.2 ± 3460.4 (5400–19,800)	9188.6 ± 3248.2 (5630–19,400)
2	219.9 ± 32.4 (153–266)	293.1 ± 38.5 (207–351)	22.0 ± 4.1 (16.6–29.9)	9730.0 ± 2168.6 (5940–16,900)	9766.9 ± 2135.6 (6010–16,600)
3	196.2 ± 42.2 (130–268)	448.2 ± 33.9 (351–491)	21.5 ± 2.7 (16.8–28)	10,639.4 ± 2336.6 (5680–15,600)	10,872.7 ± 2186.6 (6300–15,600)
4	20.2 ± 7.3 (0.7–53.3)	548.9 ± 59.4 (409–609)	6.2 ± 2.3 (1.8–11.4)	2772.1 ± 1318.7 (1000–4900)	3520.6 ± 1414.3 (1650–5600)
5	123.5 ± 18.9*** (63.6–138)	358.3 ± 40.5** (411–522)	17.2 ± 1.3*** (14.2–19.2)	8032.9 ± 2174.9*** (5290–13,800)	8268.6 ± 2110.0*** (5710–13,900)
6	127.4 ± 40.0*** (19–183)	358.3 ± 40.5*** (265–405)	15.2 ± 2.4*** (5.8–18)	6854.1 ± 1379.2*** (3810–10,100)	6743.7 ± 1193.0*** (3830–9550)
7	81.0 ± 16.7*** (58.2–110)	263.5 ± 50.4*** (206–339)	12.3 ± 2.0*** (8.2–15.9)	5759.1 ± 1233.4*** (3430–7850)	5636.6 ± 1081.0*** (3610–7420)

*SD* standard deviation.

Blood flow direct impinging center is on line 4. **P < 0.01 and ***P < 0.001 represent significant differences in hemodynamic parameters between lines 1 and 7, lines 2 and 6, and between lines 3 and 5.

Sphere sampling analysis of hemodynamic parameters on the aneurysm dome and at the aneurysm initiation location following virtual aneurysm removal (Fig. 1C1, C2 and Table 6) demonstrated that the dynamic pressure (23.1 ± 0.6 vs. 249.2 ± 5.3 Pa), total pressure (532.7 ± 1.2 vs. 743.7 ± 8.1 Pa), vorticity magnitude (2961.9 ± 33.9 1/S vs. 11182.7 ± 209.7 1/S), WSS (5.3 ± 0.1 Pa vs. 23.4 ± 0.3 Pa) and strain rate (3105.2 ± 33.5 1/S vs. 11142.6 ± 199.2 1/S) were all significantly (*P* < 0.0001) decreased on the aneurysm dome compared with those at the aneurysm initiation site.

**Table 6:** Hemodynamic stresses on the aneurysm dome and initiation site after virtual aneurysm removal.

Hemodynamic parameters	Aneurysm dome	Aneurysm initiation site
Dynamic pressure (Pa)	23.1 ± 0.6*** (0.26–138.3)	249.2 ± 5.3 (175.1–337.8)
Total pressure (Pa)	532.7 ± 1.2*** (431.3–619.4)	743.7 ± 8.1 (639.0–884.3)
Vorticity (1/S)	2961.9 ± 33.9*** (758.1–8971.8)	11,182.7 ± 209.7 (7583.2–17,671.1)
WSS (Pa)	5.3 ± 0.1*** (0.9–16.6)	23.4 ± 0.3 (18.2–28.5)
Strain rate (1/S)	3105.2 ± 33.5*** (641.0–8838.9)	11,142.6 ± 199.2 (7795.7–17,760.5)

***P < 0.0001, significant compared with aneurysm initiation site after virtual aneurysm removal.

## Discussion

It has been reported that the aneurysm initiation site most consistently coincided with high WSS and WSS gradients^12,16–19^, and combination of high WSS and positive WSS gradients in the adjacent region of flow acceleration triggers pathological remodeling and subsequent aneurysm formation^4,20^. Based on our previous findings that most ACA and BA aneurysms deviated to the smaller lateral angle and smaller daughter branch^6,7^, we hypothesized that formation of cerebral aneurysms was associated with both arterial bifurcation geometry and hemodynamic stresses, particularly the arterial branch forming a smaller lateral angle with the parent artery. It has been reported that the region enclosed by the aneurysm neck was the “aneurysm initiation site”^12^. However, the direct flow impinging center which is enclosed by the aneurysm ncek has the least WSS and is consequently not the site of aneurysm initiation^8,12^. The aneurysm initiation site is very small and is probably in the flow accelerating region adjacent to the direct flow impinging center, where pathological remodeling is initiated by abnormally-enhanced hemodynamic stresses for aneurysm formation^4,20^. In our study, we explored the possible location of aneurysm initiation by investigating aneurysm deviation towards the smaller lateral angle and smaller branch and magnitude of hemodynamic stresses on the bifurcation apex at four major cerebral arterial bifurcations. We found that Peak 1 on the branch forming a smaller lateral angle with the parent vessel had significantly greater hemodynamic stresses than Peak 2 on the contralateral side and was probably the aneurysm initiation site. Our study showed that most aneurysms at the four major cerebral arterial bifurcations deviated towards the smaller lateral angle and smaller branch, which confirmed previous finding that most ACA and BA aneurysms deviated toward the smaller branch and smaller lateral angle^6,7^. This may indicate that a smaller-diameter branch with a smaller lateral angle formed between the branch and the parent artery may induce abnormally-enhanced hemodynamic stresses to initiate an aneurysm. It was further found that 65.2%, 80%, 64.4% and 90.8% smaller lateral angles were formed between the smaller branch and the parent artery in ACA, ICA, BA and MCA bifurcations, respectively, significantly more than those formed between the larger branch and the parent artery (*P* < 0.001). Because bifurcation angle changes are associated with significant hemodynamic stress alterations on the bifurcation apex^8,13,21^ and aneurysmal MCA bifurcations tend to be asymmetrical^22^, aneurysm initiation is associated with asymmetrical arterial bifurcation structure, including asymmetrical lateral angles and branches, smaller lateral angles and smaller branches inparticular, which may consequently result in abnormally-increased hemodynamic stresses on the bifurcation apex to initiate an aneurysm.

Sampling analysis of the hemodynamic parameters in the longitudinal line on the bifurcation apex wall with virtual aneurysm removal demonstrated that the total pressure was the greatest but the other parameters (dynamic pressure, WSS, vorticity and strain rate) were the least at the direct flow impinging center, which confirmed previous findings^8,10,11,23^. As blood flowed distally towards branching arteries, the total pressure decreased quickly while the other hemodynamic parameters increased rapidly to reach the peak at the branching arteries. Peak 1 with greater hemodynamic stresses was on the same side as aneurysm deviation and was also on the smaller lateral angle side when using the parent vessel centerline as the reference. Seven transverse lines were drawn on the bifurcation apex for sampling hemodynamic stresses, with lines 3–5 for sampling the direct flow impinging center and two peaks while lines 1, 2, 6 and 7 for sampling places further down the branch in order to analyze changes of hemodynamic stresses with distance. Comparison of the hemodynamic stresses at these lines was performed to find the location with significantly different hemodynamic stresses and possible aneurysm initiation site. Transverse lines 1 and 7 were located on the vessel wall outside of the aneurysm, indicating that these areas with their characteristic hemodynamic stresses were not the aneurysm initiation site. Line 4 was located at the flow direct impinging center and has the hemodynamic characteristics of maximal total pressure but least dynamic pressure, WSS, vorticity and strain rate. Lower WSS can only induce artery intimal thickening rather than destruction^24^ and thus, line 4 could not be the location of aneurysm initiation. Transverse lines 2, 3 and 5, 6 were located within the aneurysm scope and were possibly the site of aneurysm initiation. The mean total pressure, dynamic pressure, WSS, vorticity and strain rate on lines 2 and 3 were significantly greater (*P *˂ 0.001) than those on lines 5 and 6, respectively, which probably indicates that the hemodynamic stresses characteristic of the region around line 3 or between lines 2 and 3 on the smaller branch are great enough to initiate an aneurysm. Line 3 indicated Peak 1 in the accelerating region adjacent to the direct flow impinging center and may consequently be the site of aneurysm initiation. After aneurysm formation, the hemodynamic parameters (dynamic and total pressure, WSS, vorticity and strain rate) on the aneurysm dome were significantly decreased compared with those at the aneurysm initiation site following virtual aneurysm removal. This indicates that aneurysm formation is to decrease the abnormally-enhanced hemodynamic stresses on the vascular wall so as to maintain these stresses within a certain physiological range^8,25,26^.

At the bifurcation apex, the flow is perpendicular to the apex wall and is divided at the flow impinging center. As flow moves rapidly towards branches, the hemodynamic stresses other than the total pressure reach their peaks before the flow becomes laminar with the flow direction parallel to the arterial wall. The direct impingement center of flow and its adjacent region up to the peak site are the flow acceleration region where turbulent flow forms vortices and eddies to injure the arterial wall^8^. At the arterial bifurcation apex, some densely packed ligaments exist to protect the bifurcation apex from being damaged by abnormally-increased hemodynamic stresses caused by turbulent flow^8,27^. On the branch forming a smaller angle with the parent artery, the blood flow takes a greater distance to become laminar, and the peak site of hemodynamic stresses was located farther downward beyond the protection of these densely packed ligaments at the bifurcation apex. Consequently, the hemodynamic stresses at the peak site on the smaller branch forming a smaller angle with the parent artery will damage the arterial wall and induce an aneurysm. At the direct impingement center of flow, the kinetic energy carried by the rapidly moving blood flow will be converted to the potential energy of pressure after the flow hits the bifurcation apex, resulting in the maximal total pressure at the direct impinging center. Because the speed of flow is decreased to the minimal at the direct impinging center of flow, the other hemodynamic stresses like WSS, dynamic pressure, vorticity and strain rate are also significantly decreased. In the direct impinging center, the blood flow direction is perpendicular to the wall; when the blood flow moves away from the direct impinging center, the blood flow is sped up and becomes laminar where the flow direction is parallel to the branch arterial wall. As the flow speed increases, the total pressure decreases while the other hemodynamic stresses (WSS, dynamic pressure, vorticity and strain rate) are increased quickly to the peak values (Peak 1 and Peak 2) in the region immediately adjacent to the direct impinging center. At the direct flow impinging center, the shear stress is the least which can only induce intimal hyperplasia and wall thickening^24,28^, and the direct impinging center is consequently not the site of aneurysm initiation. At the peak site especially Peak 1, the WSS, dynamic pressure, vorticity and strain rate are the maximal, whereas the total pressure remains very high. High shear stress will damage the vascular wall endothelial cells and predispose the artery to destructive aneurysmal remodeling^29–31^. After arterial destructive damage at the peak site, concomitant high pressure at this site will cause the arterial wall to expand outward to form an aneurysm. Consequently, pressure, shear stress and other hemodynamic stresses work together to initiate an aneurysm^8^.

Based on this analysis, some measures can be taken clinically to better treat cerebral aneurysms. For example, stenting can be used as a major measure rather than just a tool for assisting embolization of cerebral aneurysms. Gao et al. have studied the effect of stenting with one or two stents in assisting embolization of cerebral aneurysms at arterial bifurcations^9–11^. Stent deployment at arterial bifurcations can significantly increase the stented lateral angle, decrease the bifurcation angle, displace and attenuate the flow impinging zone, and decrease the WSS and total pressure at the bifurcation apex. The attenuation of hemodynamic stresses on the bifurcation apex caused by stent deployment may decrease the stresses for aneurysm recurrence. Recent CFD studies investigating hemodynamic stresses for aneurysm recurrence supported this theory, and recurrent aneurysms had greater WSS and flow velocity besides formation of small vortex at the aneurysm neck than non-recurrent aneurysms^32–35^. To be specific, stenting can displace the site of aneurysm initiation according to our study and decrease the abnormally-enhanced hemodynamic stresses and subsequent wall damage on the branch. With displacement of aneurysm initiation site and decrease of hemodynamic stresses, the rate of aneurysm recurrence and further expansion is consequently decreased, leading to better clinical outcome. Thus, a better approach for treating cerebral aneurysms at arterial bifurcation is to deploy a stent into the branch artery forming a smaller angle with the parent artery so as to enlarge the smaller lateral angle, displace the site of aneurysm initiation and decrease hemodynamic stresses and subsequent aneurysm recurrence.

In our study, we used some virtual techniques to remove the aneurysm for restoring the arterial bifurcation morphology to the state before aneurysm formation. Virtual removal of cerebral aneurysms has been applied by many authors in the cerebral aneurysm research for investigating hemodynamic stresses before and after aneurysm formation^8,10,11,23,36–38^, including researchers from our lab^8,10,11,23^. This is because a saccular cerebral aneurysm is only a localized pathological outpouching of a weakened arterial wall with local disruption of elastic lamina, apoptosis of medial smooth and inflammatory cell infiltration^39,40^. A saccular cerebral aneurysm only affects a small part of the vessel wall in pathology and is quite different from a fusiform aneurysm which involves the entire arterial wall along a certain distance like a deformed cigar^40,41^. Therefore, virtual removal of the local aneurysm will not affect the whole bifurcation morphology and can reflect the status of the arterial bifurcation before aneurysm formation.

In our study, we did not evaluate pathological changes at the aneurysm initiation site, namely, the Peak site of hemodynamic stresses, and the adjacent region at the bifurcation apex. Analysis of the pathological changes at these sites may add potent evidence for aneurysm initiation. Moreover, this study was limited to the Chinese population without involvement of other ethnicities or races. Interpretation and generalization of the results should be cautious to other races or ethnicities. These may be the limitations of this study and have to be solved in the future work.

In summary, the bifurcation arterial branch forming a smaller angle with the parent artery may cause abnormally-enhanced hemodynamic stresses to initiate an aneurysm on the bifurcation apex.
